# Supplementary material for: Bioinformatics analysis of the potentially functional circRNA-miRNA-mRNA network in breast cancer
Source: PLoS One. 2024 Apr 18;19(4):e0301995. doi: 10.1371/journal.pone.0301995 (PMC11025867; doi:10.1371/journal.pone.0301995)
Supplement: S1 File — (DOCX) [file pone.0301995.s001.docx]

**Supporting Figures**


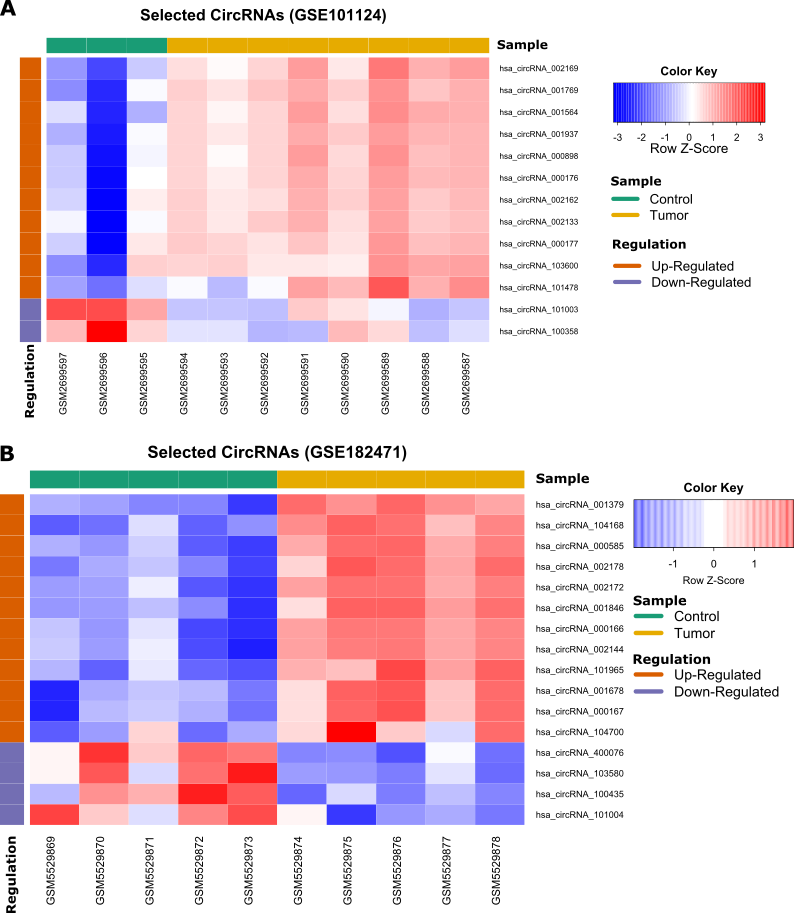


**S1 Fig.** Heatmap for the overlapped up- and down-regulated DECs for each microarray datasets, A: GSE101124, B: GSE182471. The heatmap was created by R package ‘gplots’. DECs: differently expressed circRNAs.


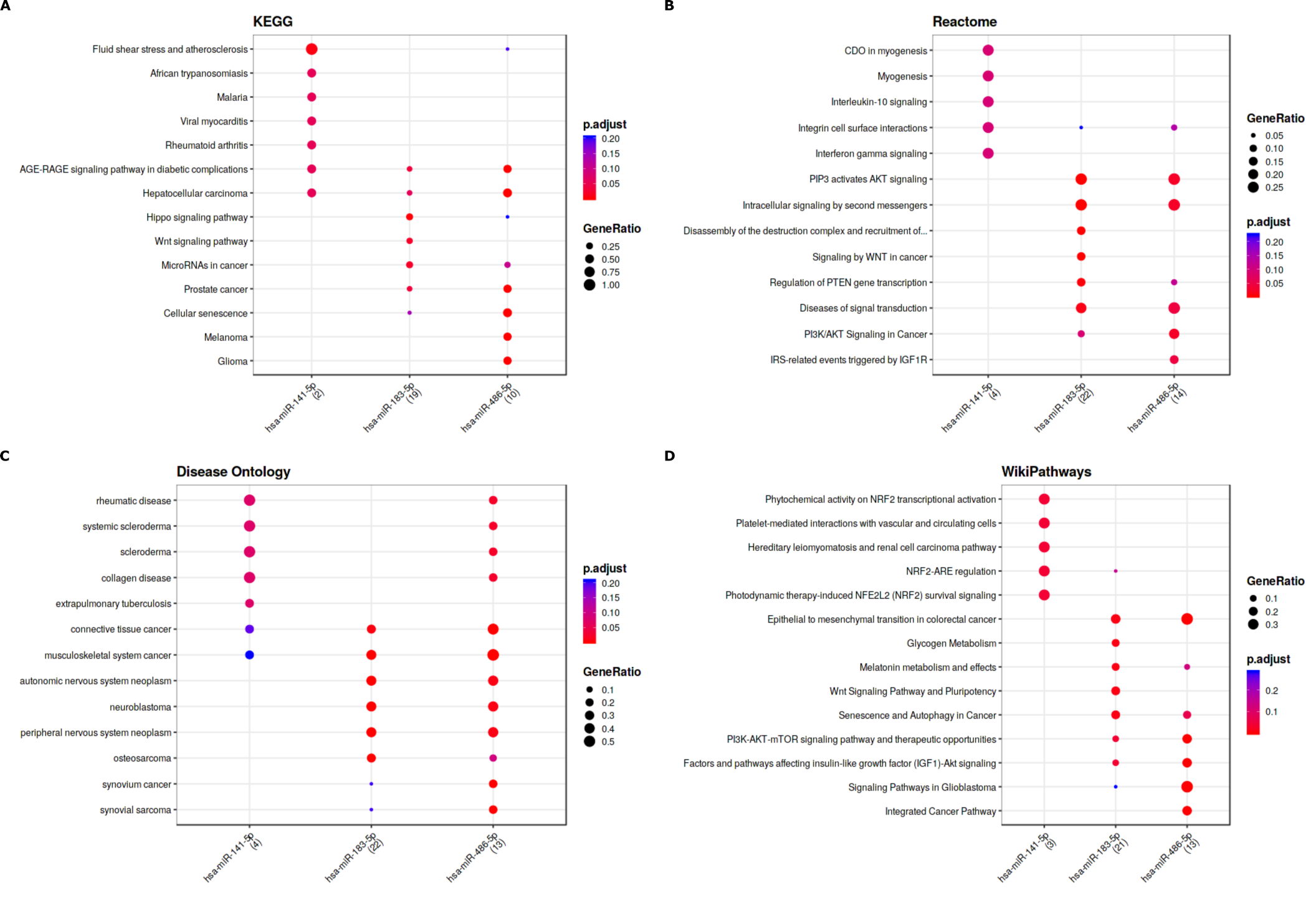


**S2 Fig.** Enrichment analysis results for the significant signaling pathways that the three miRNAs (*miR-486-5p*, *miR-141-5p*, and *miR-183-5p*) related according to the MIENTURNET (http://userver.bio.uniroma1.it/apps/mienturnet/). A: KEGG pathways, B: Reactome pathways, C: Disease Ontology, D: WikiPathways.


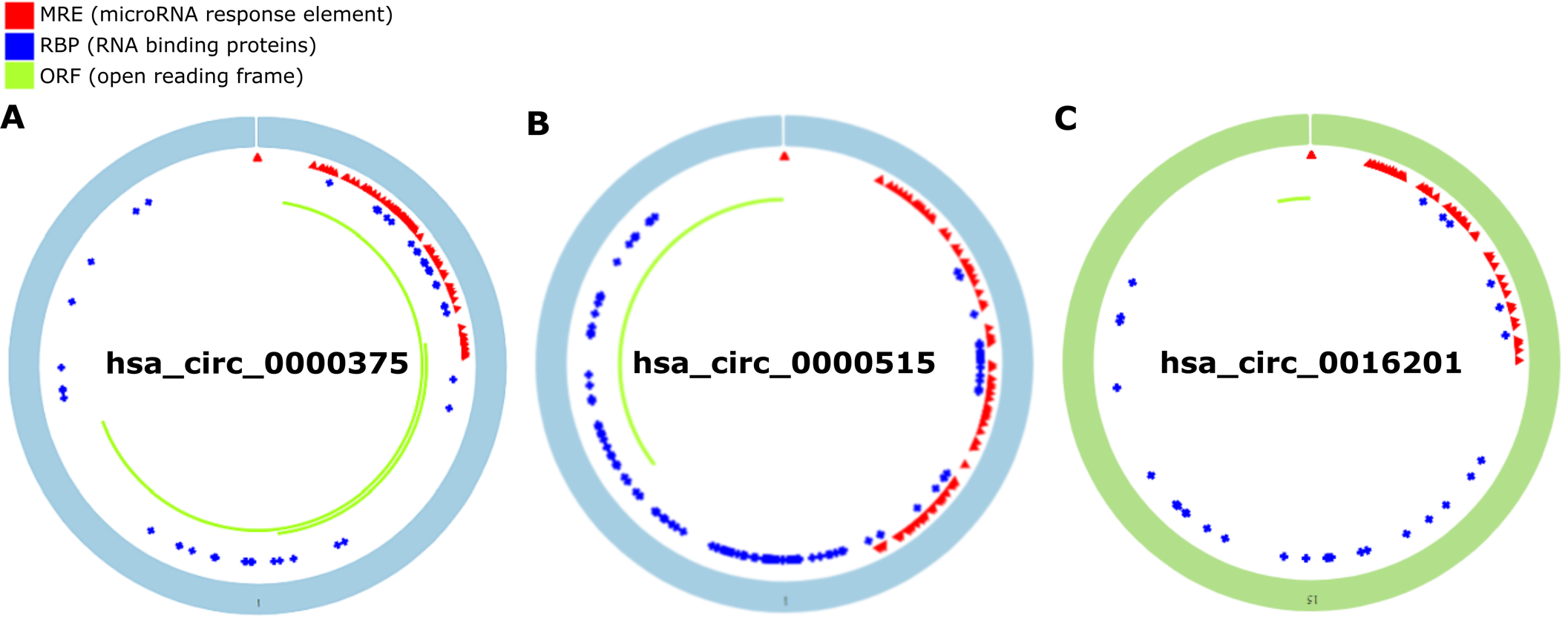


**S3 Fig.** Structural patterns of the three DECs by the Cancer-Specific CircRNA (CSCD v2.0, http://geneyun.net/CSCD2/), a: *has_circRNA_0000375*, b: *has_circRNA_0000515*, c: *has_circRNA_0016201*. DECs: differently expressed circRNAs.


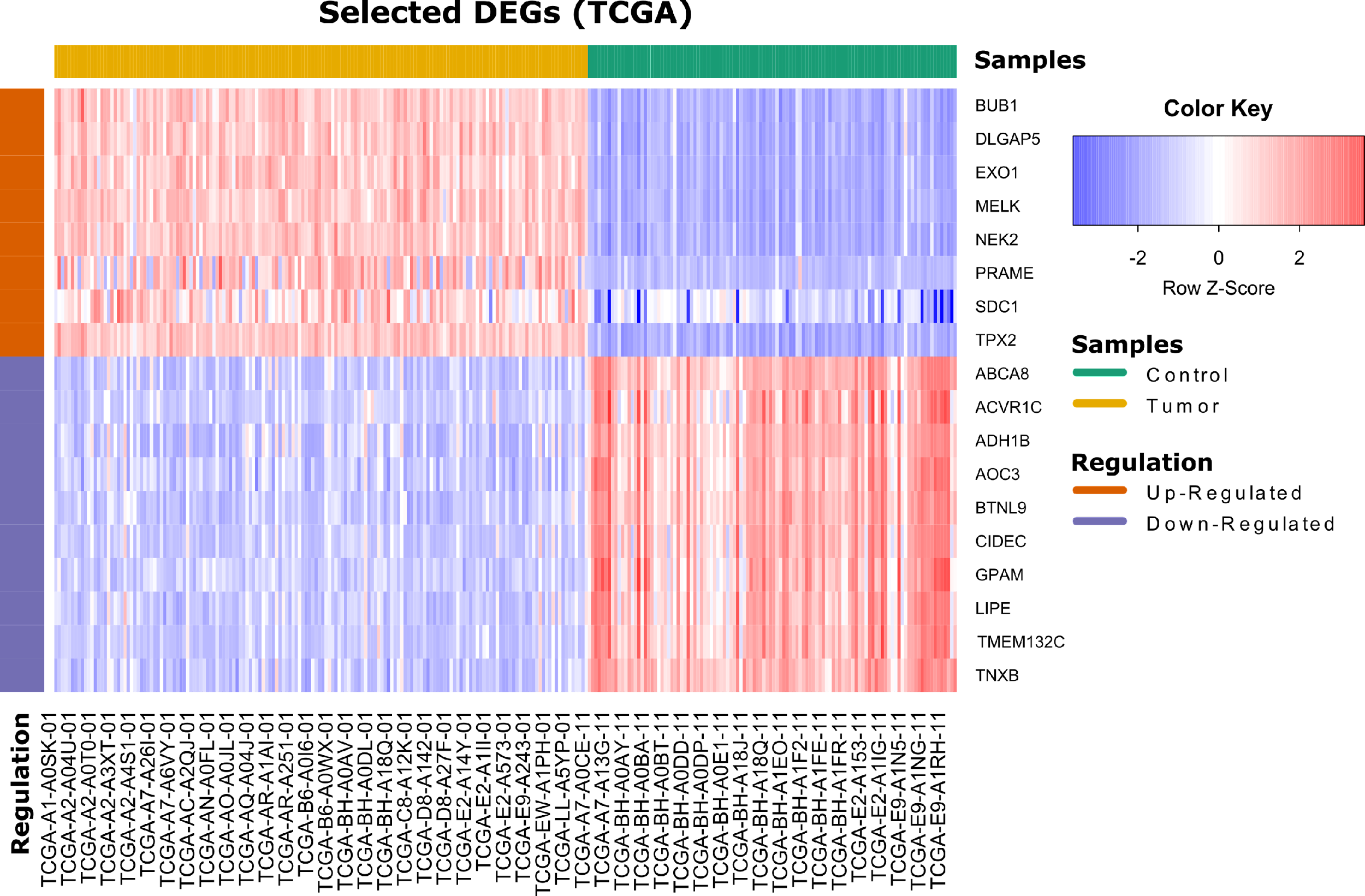


**S4 Fig.** The heatmap of 18 selected differentially expressed genes in BLBC versus control group.

**
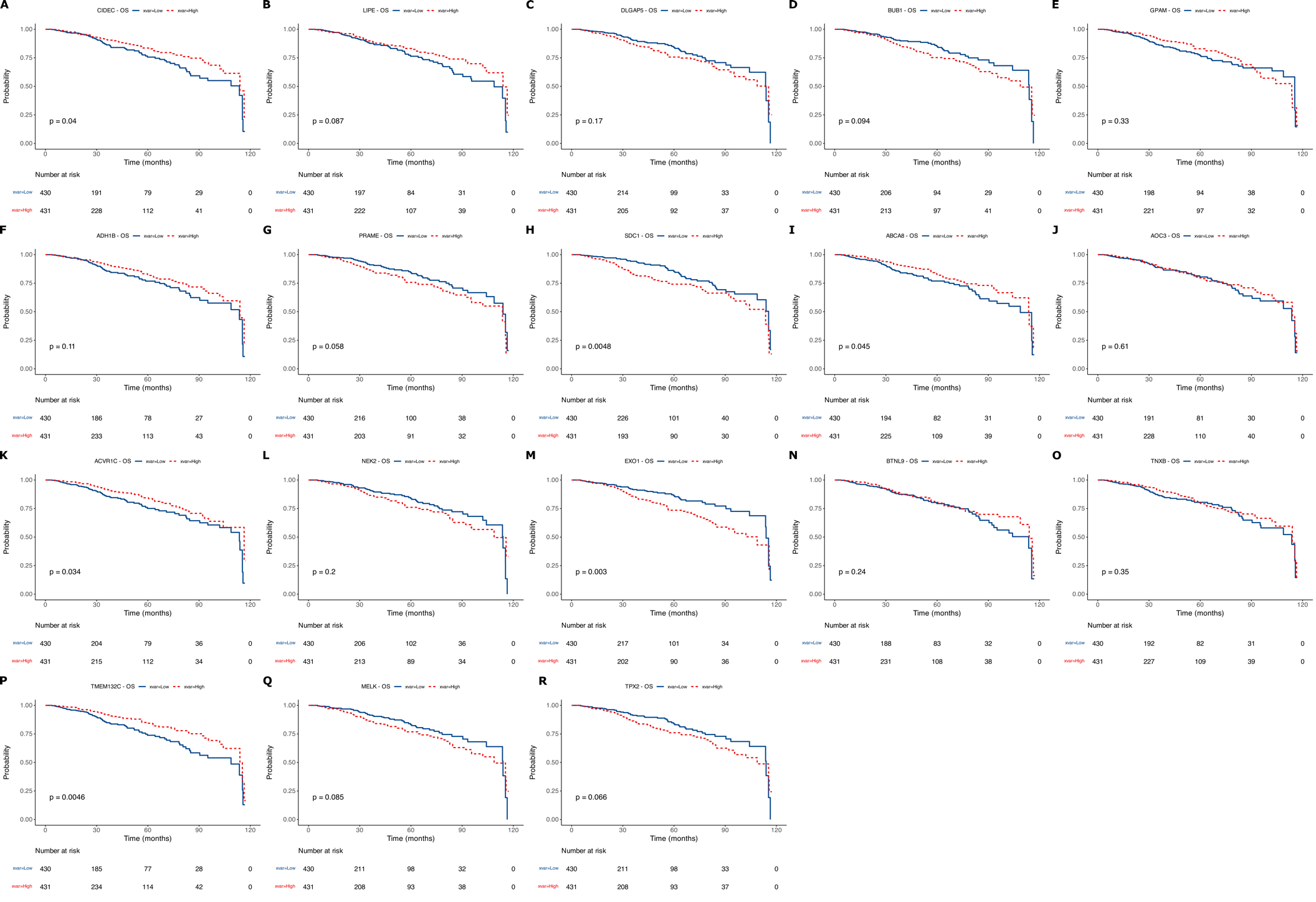
**

**S5 Fig.** Overall survival analysis results of 18 selected genes (TCGA).


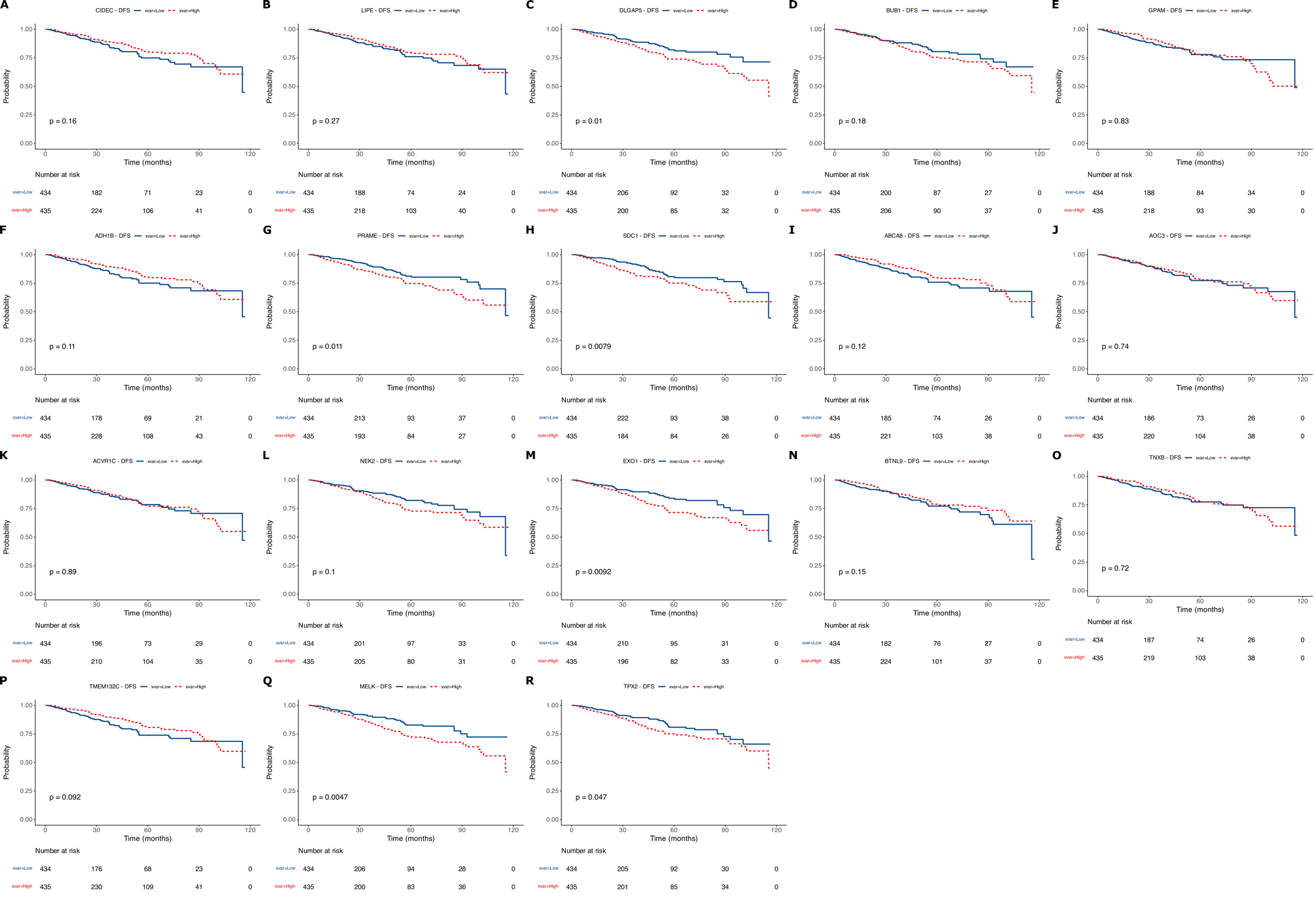


**S6 Fig.** Disease-free survival analysis results of 18 selected genes (TCGA).


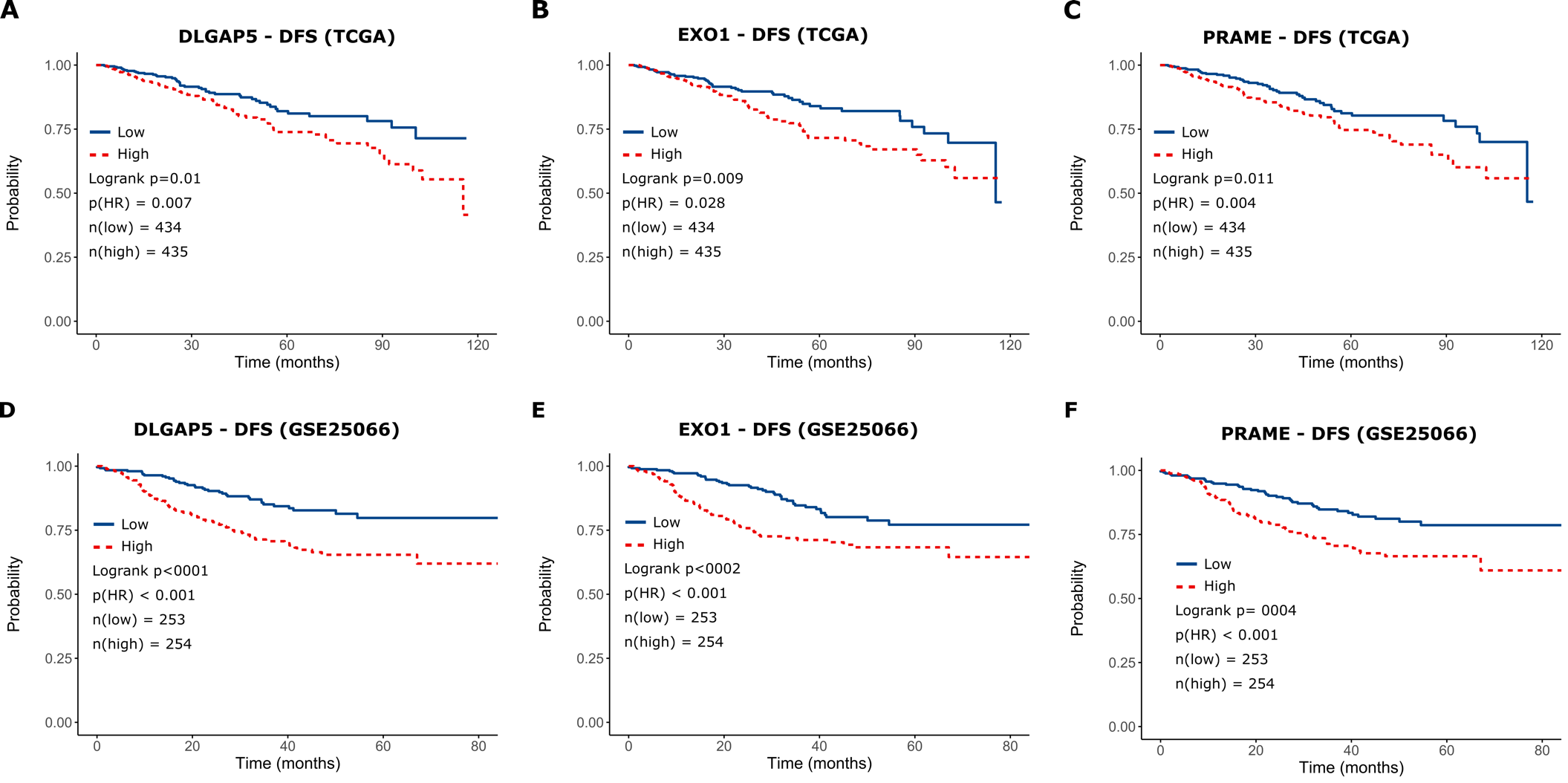


**S7 Fig.** Disease-free survival analysis of selected genes from TCGA and GSE25066 datasets. A: *DLGAP5* (TCGA), B: *EXO1* (TCGA), C: *PRAME* (TCGA), D: *DLGAP5* (GSE25066), E: *EXO1* (GSE25066), F: *PRAME* (GSE25066). DFS: Disease-free survival.


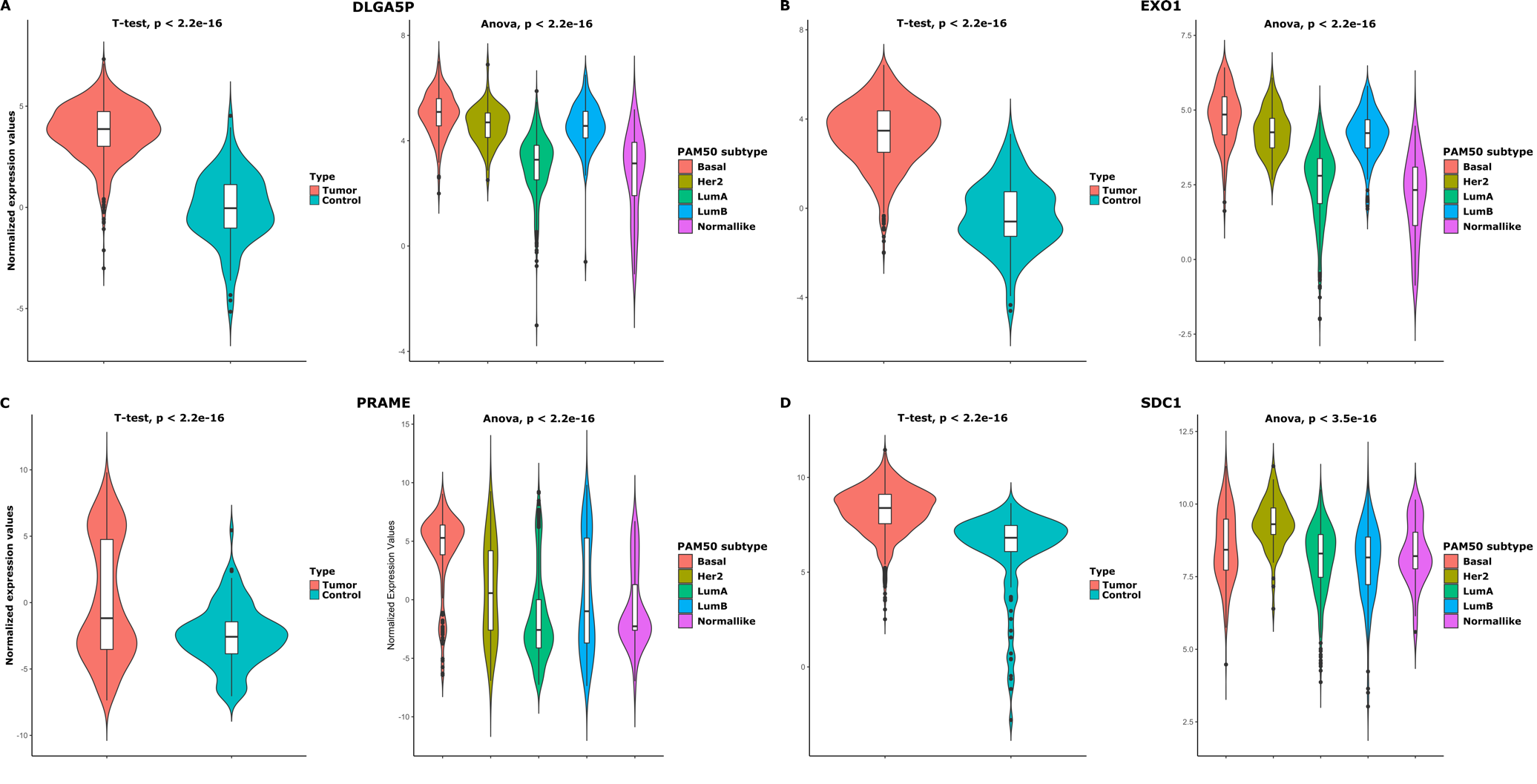


**S8 Fig.** The combined violin and box plots for the normalized expression values of selected genes in tumor versus normal tissue and PAM50 subtypes. A: *DLGAP5*, B:*EXO1*, C: *PRAME*, D: *SDC1*.
