# Supplementary material for: Bioinformatics analysis of the potentially functional circRNA-miRNA-mRNA network in breast cancer
Source: PLoS One. 2024 Apr 18;19(4):e0301995. doi: 10.1371/journal.pone.0301995 (PMC11025867; doi:10.1371/journal.pone.0301995)
Supplement: Attachment — Submitted filename: Refree evaluation..docx [file pone.0301995.s004.docx]

**Thank you for submitting your article to Plosone journal. Your article has been evaluated by the referees and the comments of the referees are given below.**

Reviewer 1

In this manuscript the authors aimed to find out regulatory networks of circRNAs, miRNAs and mRNAs in breast cancer. The manuscript has an importance since finding out circuits and networks is important to understand the development and progression of breast cancer from systems biology approach. The authors have to be more precise about the hypothesis they suggested; it can not be extracted from the manuscript whether they are dealing with the networks related to the formation (T/N) or the molecular classification (PAM50) of breast cancer. And the authors should consider the points raised below to be accepted;

The authors need to revise the word “database” to data sets” in the following sentence, which is in the introduction section of the manuscript; “Therefore in our study circRNAs that may be relevant to BC were detected using the GSE101124 and GSE182471 databases.” These series are the datasets submitted to GEO database.

Although the main aim of the study is defined as to find out the regulative circRNA-miRNA-mRNA
network in breast cancer, they do not mention anything about these networks or the structure of the network or the significance of finding out networks in the introduction. Introduction is too brief and not so informative at all.

The authors have to give the details of the selection criteria of the expression data of circRNAs. Why did they use only two array data instead of adding NGS data to increase the sample number. They may explain this as a limitation of the study.

One of the data set GSE101124 is comprised of both breast cancer cell lines and breast tumors with different subtypes (Luminal A and TNBCs). And the subtypes of the tumor samples are not mentioned in the study of GSE182471. The authors need to explain the characteristics of the data they used in the manuscript rather than to give it up to the readers. As far as it is understood the authors analyzed the cell line data together with the tumor data which is completely irrelevant. They have to exclude the cell line data since their genetic makeup is completely different from the tumor samples as they are cultured, and many passages are performed.

As it can be extracted from the figure 2 but not from the text, the authors found out the differentially expressed circRNAs among all groups of tumors compared to control samples (tumors (LumA, TNBC, cell lines) vs controls) but although they found out DE miRNAs between all subtypes they only concentrated on the basal tumors and controls. Then they related these DE miRNAs and circRNAs, which are obtained from different sample types. The authors must explain the selection criteria of the samples to be compared clearly. Otherwise, the relationship they are trying to establish between circRNAs and miRNAs could not be robust.

The selection criteria of 3 DE circRNAs among 16 common DE circRNAs for further studies is not justified. The authors have to be more specific about the biological relevance of why they selected these three. And the same comment is valid for the selection of DE 3 miRNAs. They need to explain it in the results section more clearly.

As far as I understand the authors selected DE genes by comparing all subgroups of BC to normal samples. They do not concentrate on any of the subtypes like basal like subtype. So the DE circRNAs were selected from the list of circRNAs DE between all subtypes compared to control samples like DE genes. But the DE miRNAs were selected from the list of DE miRNAs from the comparison of basal like subtype vs normal controls. This selection criteria could affect the network analysis since the elements (circRNA-miRNA-mRNA) of the network are selected from different subgroups.

The place of the Figure 3 is confusing, and it would be better to present it after Figure 5. Otherwise, it is not compatible with the flow of the text.

Reviewer 2

Circular RNAs (circRNA) are involved in regulating proliferation, invasion, and migration of cancer and may serve as novel molecular markers for diagnosis, prognosis evaluation and treatment of cancer. The current study represents a bioinformatic-based analysis to identify potential circRNA-based prognostic and targeted therapy strategies for Breast Cancer (BC). Here, the authors indicated that hsa_circ_0000515/ miR-486-5p/ SDC1 axis may be an important potential biomarker for BC patients in the basal-like breast cancer (BLBC) group. The overall level of the paper is good and important issues are emphasized. However, some issues listed below need to be addressed by the authors to enhance their manuscript.

1. Authors are requested to care using full expression of some terms followed by its abbreviation in the paper.

2. To identify differentially expressed circRNAs (DECs), Authors downloaded two datasets containing circRNA expression profiles in BC patients from Gene Expression Omnibus (GEO) database. Authors are requested to give related links or references for databases or other web-based tools (for example: CSCD v2.0) that are used in the bioinformatic analysis in the materials and method section.

3. The cutoff parameters should be given for STRING analysis in the “Analysis of the protein–protein interaction (PPI) network” title of the materials and methods section.

4. The authors found differentially expressed circRNAs (DECs) by using two independent GEO datasets (GSE101124 and GSE182471). This analysis was conducted in Breast Cancer Compared to Adjacent Tissues. However, Identification of Differentially Expressed mRNAs and miRNA were performed in PAM50 breast cancer subtypes using TCGA and metabric datasets. Authors shared the mRNA and miRNA analysis for BLCL subtype. Each of the five breast cancer molecular subtypes vary by their biological properties and prognoses. The circRNAs that were found in the current study might have a different profile when we compared the expression profile as subtype specific. The Authors are asked why they didn’t compare TCGA and metabric datasets as tumor vs normal and whether this article belongs to breast cancer in general or to the BLCL subtype. Authors are requested to explain this issue.


5.There is a similar study in the literature which was published in 2021 (https://doi.org/10.1155/2021/1732176). This study could affect the novelty of this manuscript. The authors are requested to discuss this study in terms of its advantages and disadvantages in the discussion section.

6. Calculation of Spearman’s rank correlation coefficient between DE miRNA and DE circRNA/mRNA expression profiles could be better.

7. According to the ceRNA hypothesis, circRNA can act as effective ceRNAs depending on their abundance and cytoplasmic localization. Although circRNAs are enriched mainly in the cytoplasm, some studies on circRNAs indicate different subcellular localization. Therefore, subcellular localization analysis might be important for the hsa_circ_0000515/miR-486-5p/SDC1 axis. To support this axis, the authors can at least use a prediction tool such as Circ-LocNet for subcellular localization and coding potential analysis.

8. This study needs validation such as qRT-PCR for the potential candidates.

9. Similar to DEM and DEC, DEG results should also be represented in the fig 2.

10. Authors expressed the 16 DECs as the most significantly changed ciRCRNAs in the “2.2 Predicting the associated biological features” part of the results section. 16 circRNAs, overlapping circRNAs from 2 GEO circRNA datasets or the most significantly changing circRNA?

11. The selection strategy of the 3 candidate circRNA (has_circRNA_0000375, has_circRNA_0000515, has_circRNA_0016201) is not clear. Authors are requested to explain how to find these 3 circRNAs? Selection of 3 miRNAs is also unclear. Selection of the miRNA and mRNAs could be explained in the results according to section 2.2 in the materials and methods in detail. Authors indicated that 11 common miRNAs from TCGA and metabric miRNA data sets analysis. Selection of 3 miRNAs, 3 circRNAs and genes could be shared in a better way.

12. Metabric dataset also has mRNA expression analysis data. Authors could have also used this dataset for DEG analysis or for validation.

13. Authors have found 18 genes from TCGA Breast cancer mRNA data analysis according to their criteria. However, they showed the survival analysis results for four of them. It is not clear why only these four genes were represented.
