# Supplementary material for: Bioinformatics analysis of the potentially functional circRNA-miRNA-mRNA network in breast cancer
Source: PLoS One. 2024 Apr 18;19(4):e0301995. doi: 10.1371/journal.pone.0301995 (PMC11025867; doi:10.1371/journal.pone.0301995)
Supplement: Attachment — Submitted filename: PLOSONE Review Comment (07.02.2024).docx [file pone.0301995.s006.docx]

In the revised version of the manuscript entitled “Bioinformatics Analysis of the Potentially Functional circRNA-miRNA-mRNA Network in Breast Cancer”, authors have responded all the comments to the reviwers. In my opinion, the answers to the comments and revisions are satisfactory. The manuscript is suitable for acceptance in its current form.
